# Digital Image Correlation-Based Investigation of the Shear Performance of Connection Systems of Assembled Bamboo Scrimber–Lightweight Concrete Composite Beams

**DOI:** 10.3390/ma17133268

**Published:** 2024-07-02

**Authors:** Zhiyuan Wang, Feng Wang, Huihui Liu

**Affiliations:** School of Management Science and Engineering, Anhui University of Finance and Economics, Bengbu 233041, China; wang3202310733@163.com (F.W.); 17355206118@163.com (H.L.)

**Keywords:** bamboo, assembled lightweight concrete, composite system, shear performance, digital image correlation

## Abstract

To investigate the shear performance of assembled bamboo scrimber (BS)-lightweight concrete (LC) connection systems, three groups of nine BS-LC shear connections were fabricated in this work using BS, LC, dowels, and grout. The experimental parameters included the dowel diameter and fabrication process (cast-in-place vs. assembly). Push-out tests were conducted on the specimens, and traditional linear variable displacement transducer (LVDT) measurements and the advanced digital image correlation (DIC) technique were employed to determine performance indicators such as the cross-section slip of composite members. Subsequently, the method for calculating the shear capacity of assembled BS-LC connection systems was theoretically analyzed. The research results showed that the load-slip curves measured by DIC were highly correlated with those measured by LVDT, thus, validating the reliability of the DIC data. According to the DIC data, the variations in slip of the shear connection over the interface height were further analyzed. An equation for calculating the shear capacity of dowel shear connectors was proposed based on theoretical analysis with comprehensive consideration of the experimental indicators such as the failure mode, load-slip curve, shear stiffness, and shear capacity of the specimens. The theoretical calculation values were in good agreement with the experimental results.

## 1. Introduction

Bamboo-concrete composite (BCC) beams [1,2,3,4] are made by placing concrete with high compressive strength in the upper compression zone and bamboo with excellent tensile strength in the lower tension zone, integrating these structures into a single structural member through shear connections, thereby allowing the two materials to work together. BCC beams, as main load-carrying structural members, hold significant potential for applications in buildings and bridge structures. When the composite beam undergoes flexural deformation, relative displacement will occur at the interface between the bamboo beam and concrete flange, resulting in stress redistribution and axial shear at the interface, which will be transferred longitudinally by shear connections, thus, restraining the relative slip between the two materials. Therefore, the shear performance of shear connections will directly affect the flexural performance of the composite beam. Currently, research on the mechanical properties of BCC structures has mainly focused on bamboo-cast-in-place (CIP) concrete composite members.

Wei et al. [5,6,7] conducted four-point bending tests on BCC beams and found that the load-carrying capacity and sectional stiffness of the BCC beams significantly improved compared to unreinforced bamboo beams. Hong et al. [8] proposed a BCC bridge deck and performed push-out tests on screw shear connections, revealing that the shear capacity of the connections increased with the screw diameter. Xiao et al. [9] carried out push-out tests on six types of shear connections used for BCC beams and found that notched connections, steel mesh connections, screw connections, and prestressed dowels + notched connections were more suitable for BCC beams compared to timber-concrete composite members. Shan et al. [10] experimentally studied dowel connectors for Glubam-concrete composite beams and reported that the shear capacity of the connections increased with the dowel diameter, but at a decreasing rate. Wang et al. [11] conducted push-out tests on 12 groups of bamboo-concrete connections and timber-concrete connections, and showed that bamboo-concrete connections exhibited higher shear capacity and shear stiffness compared to timber-concrete connections.

The interface slip of BCC members is considered a crucial indicator of the shear performance of connections. Considering the increasing use of digital image correlation (DIC) [12,13,14] in civil and structural engineering fields, this experimental study used DIC to collect real time data of the interface slip of BCC beams to improve the accuracy, comprehensiveness, and convenience of slip data acquisition. The DIC measurements were verified using the conventional linear variable displacement transducer (LVDT) system to ensure the accuracy and reliability of the experimental data.

Currently, many countries, including China, have promoted the construction of assembled structures. BCC structures are a structural form highly compatible with the requirements of assembled structures. However, there has been a lack of research on assembled BCC structures. Therefore, this study designed and constructed a connection system for assembled BCC beams, followed by experimental testing and theoretical analysis to investigate its shear performance. The experimental phenomena and LVDT data were previously reported in Ref. [3]. This study primarily focused on the analysis of DIC data obtained in the later stages. The load-carrying performance of the connection system was examined in detail, and based on experimental data and observations, we further explored the method for theoretically calculating the shear capacity of the connections and derived its calculation equations.

## 2. Experimental Program

### 2.1. Material Properties

The main materials involved in the shear connection specimens of this experiment included a bamboo scrimber (BS), LC, dowels, and grout. Ten BS compression specimens with dimensions of 30 × 20 × 20 mm were prepared according to the ASTM D143-09 standard [15], with 30 mm representing the along-the-grain length. Ten tensile specimens with dimensions of 20 × 15 × 370 mm were also prepared. We found that BS had a compressive strength of 104.07 MPa, a tensile strength of 149.62 MPa, and an elastic modulus of 16.81 GPa, according to experimental measurements. Three LC cylinder specimens (φ150 × 300 mm) were prepared according to the GB/T 50081-2019 standard [16], with a concrete mix ratio of 1:0.29:0.89:1.34:0.23 (cement:water:sand:haydite:water reducer). The density of LC was measured at 1826 kg/m^3^, which was about 30% lower than that of normal concrete (approximately 2500 kg/m^3^). The compressive strength of the concrete was measured at 35.47 MPa, and the mechanical properties of the grout material were tested according to the GB/T 50448-2015 standard [17]. Three bending specimens (40 × 40 × 160 mm) and six compressive specimens (40 × 40 × 40 mm) were fabricated. The test results showed that the grout material had a compressive strength of 55.96 MPa and a flexural strength of 8.31 MPa. Three 12-mm-diameter dowels and three 16-mm-diameter dowels were subjected to tensile tests, according to the GB/T 228.1-2010 standard [18]. The test results showed that the 12-mm dowel had a tensile strength of 534.88 MPa and an elastic modulus of 209.23 GPa, and the 16-mm dowel had a tensile strength of 412.96 MPa and an elastic modulus of 202.42 GPa.

### 2.2. Design of Shear Connections

**Figure 1:**
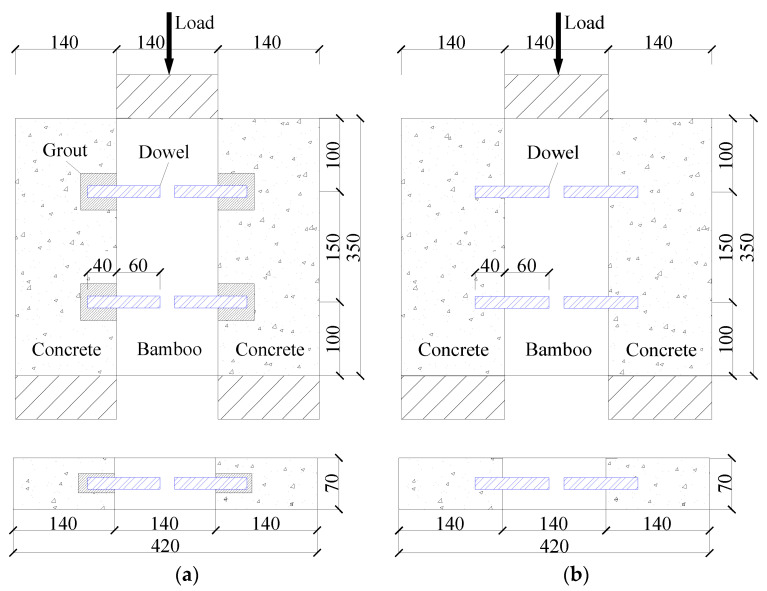
Details of push-out test of bamboo-lightweight concrete shear specimens (Units: mm). (**a**) Assembly. (**b**) Cast-in-place.

Refer to relevant literature [1,2,3,4,5,6,7,8] on bamboo-concrete connection systems, each assembled BS-LC shear connection specimen consisted of one piece of bamboo and two pieces of concrete, and its construction form is illustrated in Figure 1. The parameters of the shear connection are shown in Table 1.

The naming convention of the specimens was as follows: B = bamboo, D = dowel, C = concrete, A = assembling, and P = pouring. For example, BDC16A denoted an assembled bamboo-concrete specimen connected by 16-mm dowels, and −1/−2/−3 represented three individual specimens with the same parameters in each group.

### 2.3. Test Setup of Shear Connections

**Figure 2:**
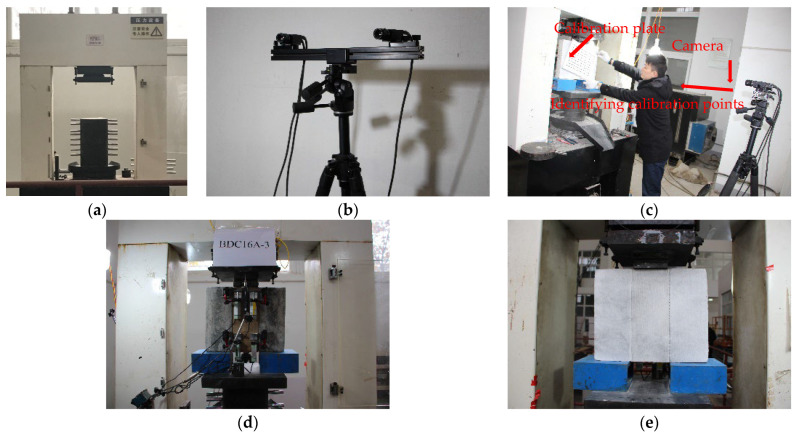
Details of the setup and measurement in push-out test. (**a**) Loading device. (**b**) DIC camera. (**c**) DIC measurement calibration. (**d**) Front LVDT device. (**e**) DIC speckle on the back.

The loading apparatus of this experiment was a 3000 kN high-stiffness rock testing machine, as shown in Figure 2. Preloading with loads of 5 and 10 kN was repeatedly applied to eliminate the influence of specimen gaps. A loading rate of 0.2 mm/min was used for formal loading in the test. When the load on the specimen exceeded the ultimate load and the load steadily decreased, the loading rate was gradually increased to 0.5 mm/min, until the test was completed.

The DIC was set up as follows. DIC measurements were conducted on the backside of the specimen. First, the specimen surface was polished smooth and cleaned. Then, white and black paints were applied to create a uniform distribution of black speckles on the specimen surface required for identification by the DIC camera. Specifically, white matte paint was first applied as a primer on the specimen surface and cured, and then sprayed with black paint. Before each test, a planar calibration plate was placed on the specimen surface for image acquisition calibration of the DIC equipment. After the calibration images passed software analysis, the test was started, and images of the specimen were captured. Two image acquisition devices were used to continuously capture images of the entire deformation process of the specimen at certain angles, where the image acquisition frequency was set to capture images every 3 s, and the relative interface slip over the entire field was calculated. The optical imaging system is shown in Figure 2. The acquisition device and VIC-3D image processing software were provided by the Correlated Solution Company in the South Carolina, USA.. The camera had a focal length of 25 mm, a resolution of 12 million pixels, and a displacement measurement progress error is 1%.

## 3. Results and Discussion

### 3.1. Load-Slip Curves and Slip Distribution

**Figure 3:**
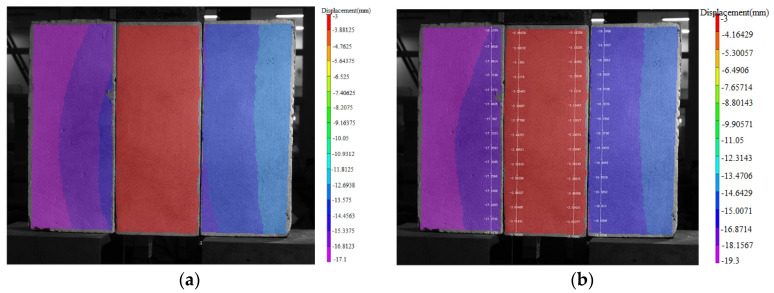
Test results and post-processing methods based on DIC. (**a**) Displacement nephogram (**b**) Analysis points near the interface.

The full-field displacement contour of the specimen surface was obtained by DIC through image processing (Figure 3).

Based on the measured displacements of the entire specimen, the relative slips between the bamboo and concrete at various positions of the specimen were determined, enabling a study of the relative slip distribution of the specimen. In this experiment, the relative slips at different heights over the interface were measured by DIC. Specifically, points were selected on both sides of the specimen interface for analysis (Figure 3). Considering the specimen height of 350 mm, 13 analysis points were selected at intervals of 25 mm on each side of the two interfaces of the specimen, resulting in displacement data for a total of 52 points for each specimen. The difference in displacements of a pair of points at the same height on the two sides of an interface was calculated to obtain 13 relative slip values for each of the left and right interfaces.

**Figure 4:**
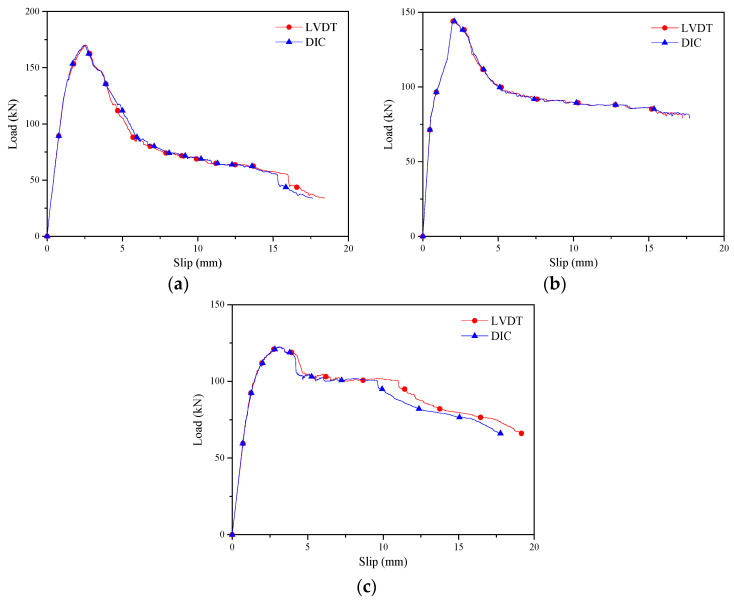
Load-slip curves of shear connections measured by two methods. (**a**) BDC16P-1. (**b**) BDC16A-2. (**c**) BDC12A-1.

Refer to relevant literature [1,2,3,4,5,6,7,8], the load slip curve was used to represent the results of the push-out test, and the test results were relatively satisfactory. Therefore, this article uses a load slip curve to represent the shear performance. Figure 4 shows several sets of representative load-slip curves measured at symmetric positions on the front and back of the shear connections by the LVDT and DIC methods, respectively. The specific measurement data is the LVDT installation position data near the pin bolt. A strong correlation between the load-slip curves by the two methods was observed. Because the LVDT measurement data were obtained from the front of the specimen and the DIC measurement data were obtained from the back, some differences were observed in the failure modes such as concrete cracking between the front and back, even though the measuring points were respectively at the same heights over the specimen surface. For example, as shown in Figure 4, specimen BDC12A-1 contained more concrete cracks on the front side, while almost no cracks were observed on the back side. As a result, the measurement data obtained by the two methods were not entirely consistent. Because LVDT failed to measure the full-field displacement data of the specimen, the slip distribution patterns investigated in this section were based on the DIC measurement data, and the traditional LVDT measurement data validated the reliability of the DIC measurement data.

**Figure 5:**
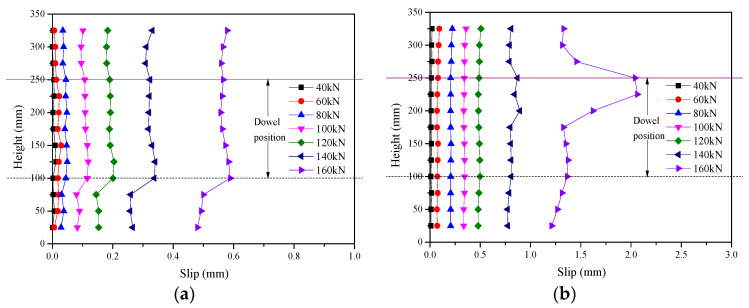

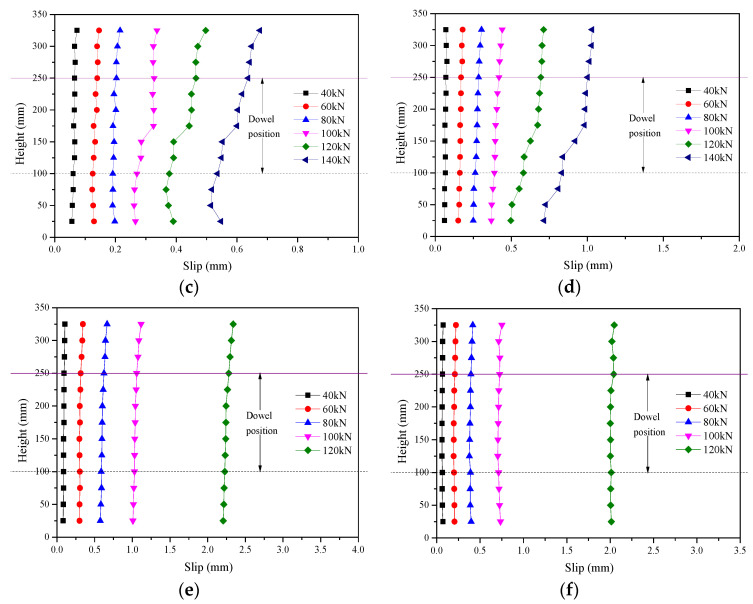
The curves of the slip of shear specimens along interface height. (**a**) BDC16P-1 Left. (**b**) BDC16P-1 Right. (**c**) BDC16A-1 Left. (**d**) BDC16A-1 Right. (**e**) BDC12A-1 Left. (**f**) BDC12A-1 Right.

To understand the interfacial slip distribution under different load levels, slip values corresponding to loads of 40, 60, 80, 100, 120, and 140 kN were obtained. The variations in slip over the interface height are shown in Figure 5.

The slip values on the left and right sides of the specimen were basically consistent, indicating no load eccentricity during the loading process before reaching the ultimate load, resulting in uniform loading on the connections of both sides. In the early stage of loading, the lines connecting 13 measurement points at different heights formed approximately straight lines without abrupt change in slip values, indicating stable force transfer of the connections. Under the same load increment, the spacing between the lines connecting 13 measurement points at different heights increased, indicating a continuous decrease in the shear stiffness of the specimen, which was related to local concrete failure and connector yield deformation. When the load approached the ultimate load, the lines connecting 13 measurement points at different heights no longer formed straight lines. In the CIP dowel specimens (BDC16P), relatively large slips were observed at the dowel positions, which, based on the experimental phenomena, were preliminarily attributed to local concrete failure at these positions. In the assembled dowel specimen (BDC16A), slips at the upper and lower dowel positions differed, but did not show abrupt changes, preliminarily indicating the presence of microcracks in the grout blocks at positions with relatively large slips. Analysis showed that the connection point of the high-strength grout block of the assembled specimens had higher strength than the LC connection point of the CIP specimens.

**Figure 6:**
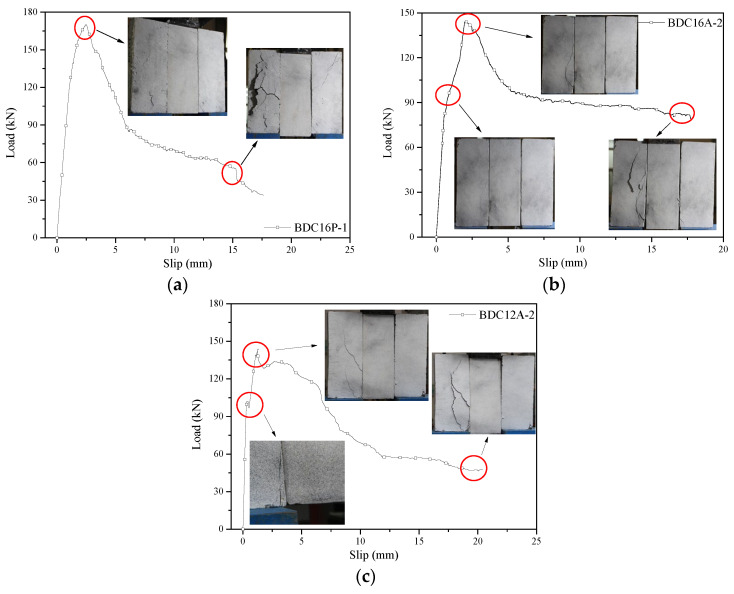
Typical failure modes of bamboo-lightweight concrete composite shear connections. (**a**) BDC16P-1. (**b**) BDC16A-2. (**c**) BDC12A-2.

The load-slip curves of the shear connections are shown in Figure 6. Each curve could be roughly divided into three stages. In the first stage, the load was approximately linearly related to the slip, indicating an effective connection of the connectors with bamboo and concrete, reliable shear transfer, and no obvious damage to the specimen. In the second stage, as the load continued to increase and approached the ultimate load, the curve still increased but with a significantly reduced slope, indicating a noticeable decrease in the rate of load increase and a rapid increase in slip. The load-slip curve also exhibited nonlinearity, primarily due to local cracking of the concrete leading to a decrease in shear stiffness. In the third stage, the curve started to descend, indicating a decrease in load, which was mainly due to local cracking and failure of the concrete, yielding deformation of the connectors and a decrease in bond strength between the connectors and the concrete. As loading continued, the load decreased slowly while slip increased rapidly. When the relative slip reached 15–25 mm, the specimen tended to tilt and separate, indicating the essential connection failure and significantly reduced capacity, therefore, the test was terminated. Specimen observation after testing revealed that the dowels remained firmly embedded in the bamboo, with penetrating cracks in the concrete from top to bottom. The ultimate failure mode of the specimens involved surface concrete spalling and varying degrees of bending of the dowels. No compressive failure occurred at the contact points between the dowels and the bamboo where the bamboo was perforated. There were no significant differences in the failure modes between the CIP specimens and the assembled specimens.

### 3.2. Flexural Stiffness and Capacity

As the shear stiffness and capacity results of the bamboo-concrete connections are shown in Table 2, indicating the shear stiffness provided by single connections. Because each dowel shear specimen had four connections, the shear stiffness provided by a single connection was ¼ of the total shear stiffness. The stiffnesses of different specimens were compared in terms of their initial stiffnesses, and calculated as 0.4 times the maximum load capacity divided by the corresponding displacement. The average values for each group of specimens were used in the calculation. According to Figure 6 and Table 2, the shear stiffness and capacity of the shear specimens showed the following trends.

(1) In the load-increasing stage, the stiffness gradually decreased.
(2) The assembled specimens had higher stiffness but lower capacity than the CIP specimens.
(3) The assembled specimens had a capacity similar to that of CIP specimens and a higher stiffness but lower ductility than the CIP specimens.
(4) In the assembled dowel specimens, the specimens with 16-mm dowels had higher capacity but lower ductility than those with 12-mm dowels. The high-strength grout locally reinforced the connecting area between the concrete and the steel plate, preventing concrete from becoming easily damaged and enhancing ductility.

## 4. Theoretical Analysis

Relatively little theoretical research has been conducted on the shear capacity of bamboo-concrete shear connections, and no theoretical research reports have been published on the shear capacity of assembled bamboo-LC shear connections. According to well established methods for calculating steel-concrete shear connections and timber-concrete shear connections, this study investigated the method for theoretically calculating the shear capacity of dowel shear connectors. The research ideas were as follows.

First, based on the force-bearing characteristics of specific connections, the shear resistance mechanism of the connections was analyzed to understand their working mode and the main factors affecting shear strength.

Second, representative equations for calculating the capacity of steel-concrete and timber-concrete connections were used to calculate the capacity of bamboo-concrete connections in this study. The calculation results were comparatively analyzed to evaluate the applicability of the relevant calculation methods. In addition, attempts were made to utilize a shear capacity calculation method suitable for bamboo-concrete connections by modifying and improving available equations based on existing theories.

Third, based on the force-bearing characteristics of bamboo-concrete connections and comprehensively considering various influencing factors, an equation for calculating the shear capacity of bamboo-concrete connections was proposed in this study.

Fourth, using the proposed equation, the shear capacity data of the specimens in this study and the shear capacity data of bamboo-concrete connections in previous studies were calculated and compared to evaluate and verify the applicability of the proposed equation.

**Figure 7:**
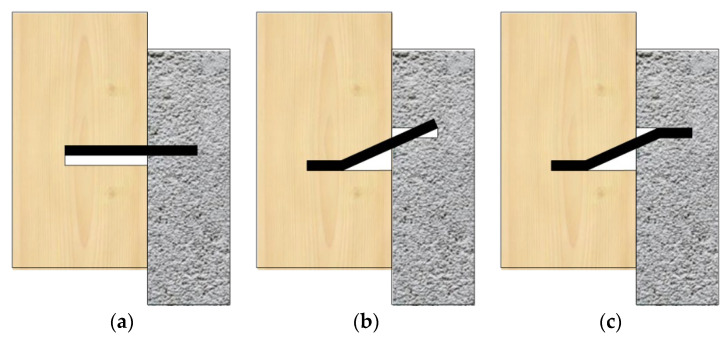
Three failure models for timber-concrete connections. (**a**) Model 1. (**b**) Model 2. (**c**) Model 3.

Dias [19] demonstrated that in timber-concrete shear connections, only three failure modes would occur (Figure 7), considering the significant confinement by concrete, namely: (1) compression failure of timber in the dowel hole, (2) formation of a plastic hinge in the timber around the connections, and (3) formation of a plastic hinge in the timber and a plastic hinge in the concrete around the connection.

**Figure 8:**
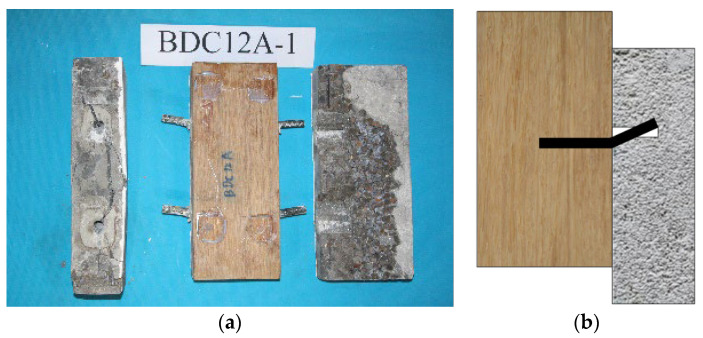
Failure model for bamboo scrimber-lightweight concrete connections. (**a**) Failure picture (**b**) Failure model.

Comparison with the failure modes of the above composite members revealed that the failure mode of the BS-LC connection in the experiment (Figure 8) shared some characteristics of the second failure mode of the timber-concrete connections (Figure 7b). Furthermore, in the experiment, only one failure mode was observed, specifically characterized by concrete cracking, dowel connector deformation, and no compressive deformation of bamboo, with significant slip deformation of the specimen before reaching the ultimate load.

**Figure 9:**
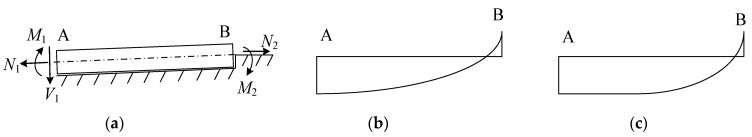
Analytical model of dowel embedded in concrete. (**a**) Pin isolation body. (**b**) Early vertical stress situation. (**c**) Post vertical stress situation. Note: A, B represents the endpoint of the dowel embedded in concrete, A is at the junction of bamboo and concrete, and B is inside the concrete; *M* represents the bending moment; *V* represents shear force; *N* represents axial force.

**Figure 10:**
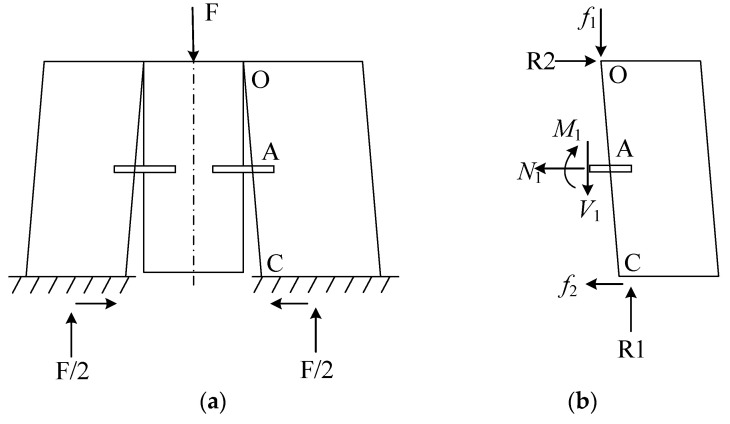
Analytical model of shear specimens in push-out tests. (**a**) Push-out specimens. (**b**) Concrete isolation body. Note: *f*_1_ represents the downward friction force generated by the upper part of the bamboo block on the upper part of the concrete. *f*_2_ represents the inward frictional force generated by the base on the lower surface of the concrete. R represents the support reaction force; F is the force; O, A and C are the three position points shown in the diagram; *M* represents the bending moment; *V* represents shear force; *N* represents axial force.

As shown in Figure 9, in the dowel shear connectors, the two ends of the dowel were subjected to forces from bamboo and concrete. Assuming the concrete as an elastic foundation [20], the force transferred from the bamboo to the dowel induced a reaction force in the concrete. Significant displacement occurred at point A of the concrete foundation, while point B was restrained and consequently experienced small displacement. As a result, the dowel underwent rotation, where the forces acting at points A and B are shown in the figure. The reaction force generated in concrete is beneficial and has a restraining effect. With continued loading, the concrete at point A entered the plastic stage, and its stress no longer increased. As the external load continued to increase, the plastic zone expanded to withstand the increasing external load until the concrete failed or the dowel yielded, when the ultimate load occurred. With an increase in external load, the cross-section slip increased, and the concrete block experienced eccentric compression under the action of the dowel (Figure 10). Eccentric compression led to the outward movement of the bottom of the specimen. The reaction R1 at the base produced a moment around point O, causing the concrete slab to rotate inward toward the specimen. This possibly led to the separation of bamboo from the concrete block, consistent with the experimental observations in the later stage of loading, where some specimens experienced separation. Furthermore, we experimentally observed that the failure mode of the specimens consisted of concrete cracking. Closer observation revealed that the concrete below the dowel at the interface was locally crushed first, similar to local compression failure. The failure phenomena included the occurrence of a plastic hinge in the dowel at this location of the interface, indicating that the dowel was subjected to a large concrete reaction at its bending point.

According to the above analysis, we concluded that the main factors affecting the shear strength of dowel connectors included material properties such as the strength and elastic modulus of the concrete, the diameter, length, and material strength of the dowel, as well as the strength of the bamboo. Therefore, the ultimate shear strength of the dowel connectors was a function of these factors. Further analysis was subsequently conducted with reference to relevant research results.

Among the equations for calculating the shear capacity of dowel connectors derived by researchers, this study selected capacity calculation equations from classic references with high citations and the latest research outcomes as well as from relevant code standards for analysis. These equations were then used to calculate the shear capacity of the specimens in this study to evaluate the applicability of these equations for calculating the capacity of dowel bamboo-concrete connections.

The theoretical equations for calculating the shear capacity of timber-concrete connections considered in this study included the equations proposed by Ceccotti et al. [21], Saulius et al. [22], Lan et al. [23], Cao et al. [24], and Wang et al. [25]. The theoretical equations for calculating the shear capacity of steel-concrete connections mainly referred to the calculation equations in Eurocode 4 [26], Chinese GB 50017-2017 [27], Chinese GB 50917-2013 [28], American ANSI/AISC 360-16 [29], Canadian CSA S16-19 [30], and the equation proposed by Oehlers et al. [31]. In addition, the equation for calculating the shear capacity of steel-timber models in Eurocode 5 [32] was also referenced.

**Figure 11:**
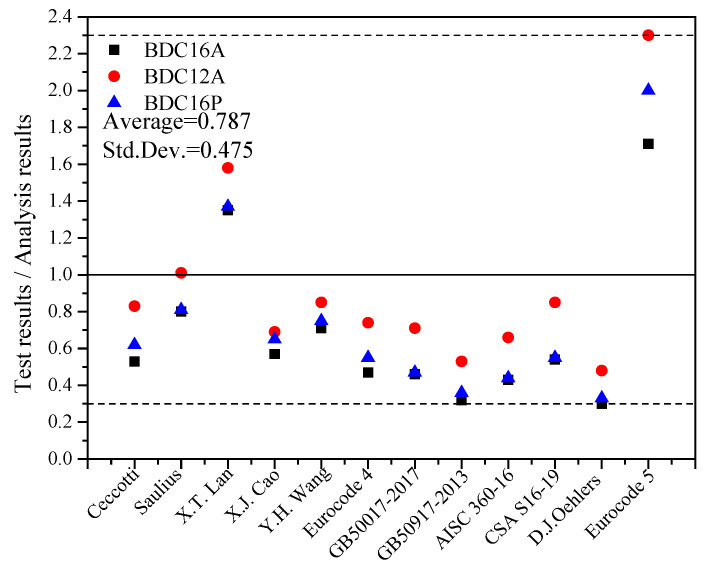
Comparison of shear capacity of bamboo-concrete connections with dowels. Note: Ceccotti corresponds to reference [21]; Saulius [22]; X. T. Lan [23]; X. J. Cao [24]; Y. H. Wang [25]; Eurocode 4 [26]; GB 50017-2017 [27]; GB 50917-2013 [28]; ANSI/AISC 360-16 [29]; CSA S16-19 [30]; D. J. Oehlers [31]; Eurocode 5 [32].

Table 3 lists some of the factors influencing the shear capacity of representative dowel connectors. In this experiment, four dowels were embedded into bamboo and concrete to form four connectors. The experimental ultimate load was divided by four to obtain the capacity of each individual connector, and the measured capacities of the specimens in this study were compared with the calculated values using the above theoretical shear capacity calculation methods, as shown in Table 4 and Figure 11. We observed that compared to the measured shear capacity of bamboo-concrete connections in this study, the shear capacity of the bamboo-concrete connections calculated using equations for dowel steel-concrete, timber-concrete, and steel-timber connections was generally larger. The ratio of measured to calculated values mostly fell between 0.3 and 0.9. Therefore, directly applying the above equations to calculate the shear capacity of bamboo-concrete connections was unsafe, indicating the poor applicability of these equations. As a result, the shear strength of the assembled bamboo-concrete shear connections could not be directly obtained using existing equations.

The existing equations were comprehensively analyzed, involving parameters such as the dowel diameter, dowel length, dowel tensile strength, longitudinal spacing of dowels, concrete strength, concrete modulus of elasticity, and the compressive strength of timber. Because the failure modes of shear connections in this experiment included the single-hinge flexural failure of dowels, failure of the concrete, and no failure of the bamboo, a summary analysis of the data calculated using the above equations revealed that the results obtained using the Saulius equation were close to the experimental results in this study. Moreover, the Saulius equation enabled reasonable mechanical interpretation and analysis, with parameters possessing clear physical meanings. In this work, further force analysis was carried out using the calculation method of the Saulius equation to derive an equation suitable for calculating the shear capacity of the bamboo-concrete connections.

The connections used in this study consisted of dowel bamboo-concrete connections, with the dowels at a right angle to the bamboo-concrete interface. According to the observed phenomena in the push-out tests, all dowel connectors experienced complete failure, and the bending mode was consistently single-hinge yield mode, without any of the three failure modes observed in the experiments conducted by Saulius et al. Preliminary analysis suggested that the reason for these phenomena was that the diameter of the dowels used in this study was larger than the diameter of the screws used in the experiments by Saulius et al., resulting in greater stiffness of the dowels. Therefore, double-hinge yield did not occur. Because the BS used in the bamboo-concrete specimens in this study had higher strength than timber, the bamboo material did not fail throughout the tests, and concrete failure only occurred at large slips; hence, no-hinge yield did not occur. Based on comprehensive analysis, a force diagram possibly applicable to the analysis of the shear capacity of the bamboo-concrete connection in this study could be obtained.

**Figure 12:**
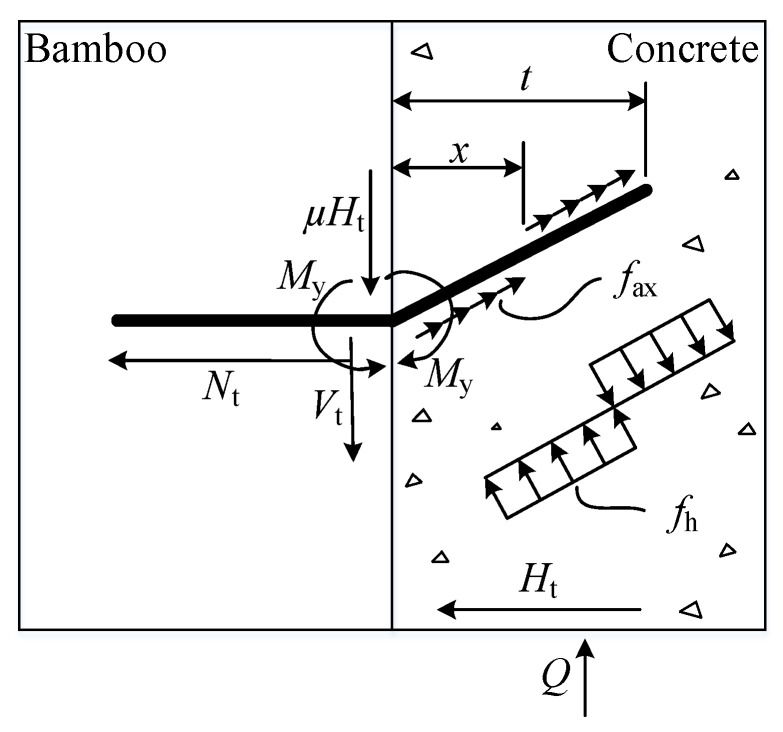
Stress analysis of single hinge yield mode of bamboo-concrete connection with dowel connector. Note: *Q* is the shear capacity of the bamboo-concrete connection, *μ* is the interface friction coefficient, *f*_h_ is the normal stress borne by the dowel in concrete, *x* is the position where the normal stress changes, *t* is the horizontal length of the dowel embedded in bamboo, *M*_y_ is the plastic hinge moment of the dowel, *f*_ax_ is the ultimate tensile strength of the dowel, *N*_t_ is the axial force on the dowel, *V*_t_ is the shear force on the dowel, and *H*_t_ is the horizontal force at the interface. Triangle is the legend of concrete; Arrows indicate the direction of force.

According to Figure 12, the force equilibrium equations were established as follows:

(1) $$Q=V_{t}+\mu H_{t},$$

(2) $$H_{t}=N_{t},$$

(3) $$N_{t}=f_{ax}\cdot d\cdot t,$$

(4) $$V_{t}=f_{h}\cdot d\cdot \left[x-\left(t-x\right)\right],$$

(5) $$M_{y}=f_{h}\cdot d\cdot x\cdot \frac{x}{2}-f_{h}\cdot d\cdot \left(t-x\right)\left(x+\frac{t-x}{2}\right),$$

(6) $$x=\frac{t}{\sqrt{2}}\cdot \sqrt{\frac{2M_{y}}{f_{h}\cdot d\cdot t^{2}}+1},$$

where *Q* is the shear capacity of the bamboo-concrete connection (N), μ is the interface friction coefficient, *d* is the dowel diameter (mm), *f*_ax_ is the axial stress borne by the dowel in concrete (MPa), *f*_h_ is the normal stress borne by the dowel in concrete (MPa), *t* is the horizontal length of the dowel embedded in bamboo (mm), *M*_y_ is the plastic hinge moment of the dowel (N·mm), *f*_u_ is the ultimate tensile strength of the dowel (MPa), *N*_t_ is the axial force on the dowel (N), *V*_t_ is the shear force on the dowel (N), and *H*_t_ is the horizontal force at the interface (N).

Because assembled specimens were used in this test, and both bamboo and concrete blocks had smooth surfaces, resulting in minimal friction between the two, the calculation equation was simplified in this study to obtain a widely applicable, concise equation by ignoring the influence of interfacial friction, i.e., taking the friction coefficient *μ* as 0. As shown in the experimental load-slip curves, the ultimate load was reached at a relatively small slip, when the bending angle of the dowel was relatively small. The large bending angle of the dowel in Figure 12 was used to emphasize the plastic hinge condition, for clarity. Considering the small bending angle of the dowel, this study used the actual length *l*_E_ of the dowel embedded in concrete to replace the horizontal length *t* of the dowel after bending. Taking into account favorable factors such as concrete confinement and interfacial friction enhancement by partial reinforcement of the concrete, a partial coefficient *φ*_sc_ was added to the corresponding equation. Rearranging the above equations, the equation for calculating the capacity of the dowel BS-LC connection in this study could be obtained as follows:

(7) $$Q=\phi_{sc}\cdot f_{h}\cdot d\cdot l_{E}\cdot \left(\sqrt{\left(4M_{y}/f_{h}\cdot d\cdot {l_{E}}^{2}\right)+2}-1\right).$$

Because the connections underwent push-out tests, the dowel in the specimen was tested only for its tensile mechanical properties, and no bending test was conducted. Therefore, the yield moment was not experimentally obtained. To determine the bending moment *M*_y_, we also referred to the calculation method proposed by Saulius et al. and calculated it based on the ultimate tensile strength *f*_u_ of the dowel using the following equation:

(8) $$M_{y}=0.8f_{u}\cdot d^{3}/6,$$

where *Q* is the flexural capacity of a single dowel (N), *φ*_sc_ is a partial coefficient, taken as 1.4, *f*_h_ is the ultimate compressive strength of concrete (MPa) *d* is the dowel diameter (mm), *l*_E_ is the actual length of the dowel embedded in concrete (mm), *M*_y_ is the moment at which plastic hinge occurs in the dowel, and *f*_u_ is the ultimate tensile strength of concrete (MPa).

**Figure 13:**
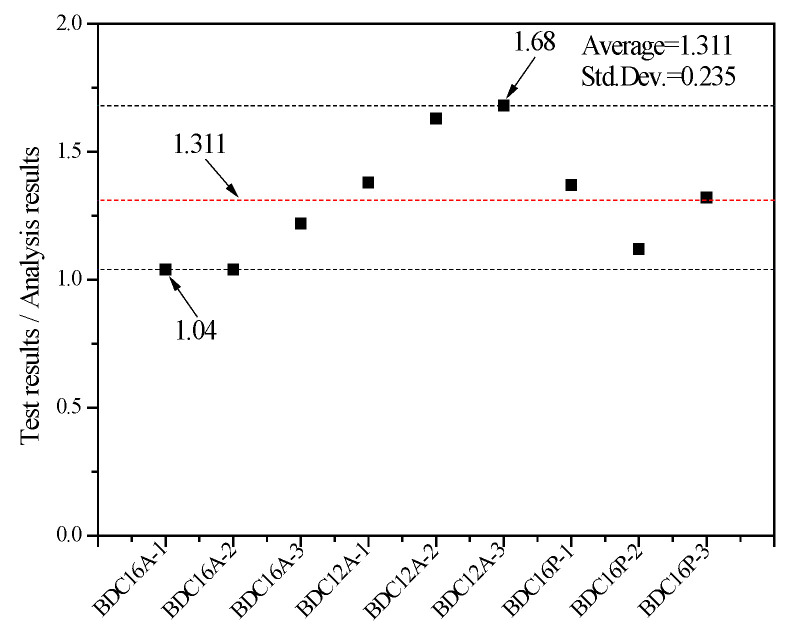
Evaluation of shear capacity of connections with dowel connectors.

The shear capacities calculated using the proposed equation were compared with the experimentally measured shear capacities in this study, as shown in Figure 13 and Table 5.

We observed that the proposed equation satisfactorily predicted the shear capacity of the specimens in this study. Moreover, the measured values are all greater than the calculated values, with the ratio of measured to calculated values ranging from 1.04 to 1.68, indicating a certain safety margin in the calculated values. However, due to the small number of specimens in this study, it was necessary to further increase the number of specimens in future research to obtain a more convincing equation through comparative analysis.

Additionally, to further verify the applicability of the equation, we collected the capacity data of relevant dowel bamboo-concrete connections from the literature [6,7,8,9,10,11] and used the proposed equation to calculate the corresponding capacities (Table 6).

**Figure 14:**
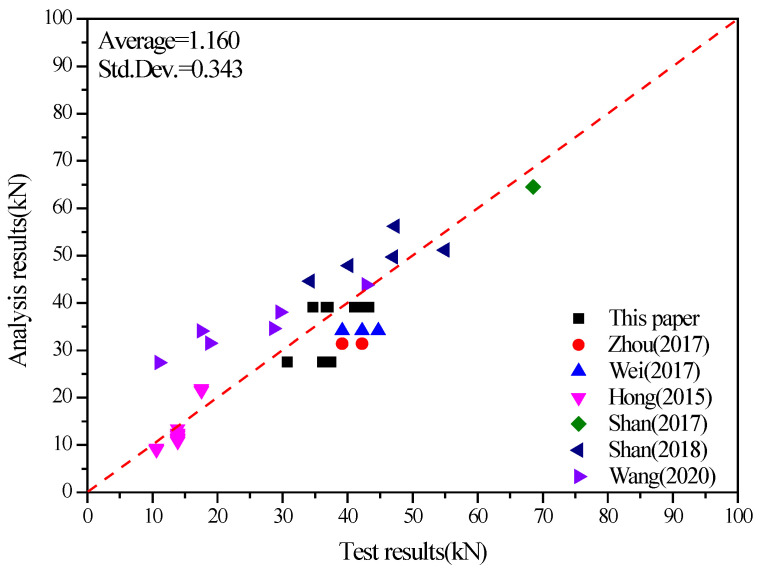
Evaluation of shear capacity equation for bamboo-concrete connections with dowel connectors. Note: Zhou (2017) corresponds to reference [6]; Wei (2017) [7]; Hong (2015) [8]; Shan (2017) [9]; Shan (2018) [10]; Wang (2020) [11].

Figure 14 shows the calculated capacities of these connections from literature using the proposed equation. The ratio of measured to calculated values had a mean of 1.160 and a standard deviation of 0.343, suggesting that the proposed equation could predict the shear capacity of dowel bamboo-concrete connections accurately.

## 5. Conclusions

This study carried out an experimental investigation and theoretical analysis of the shear performance of assembled BS-LC shear connections. In the experimental investigation, three groups of nine BS-LC shear connections with different parameters were designed and fabricated for push-out tests. Specifically, two measurement methods, LVDT and DIC, were employed, where the test parameters included the fabrication process (assembling vs. casting in place) and connection dimensions (dowel diameter). In addition, various performance indicators such as the failure mode, load-slip relationship, shear stiffness, and shear capacity of the shear connections were examined. The theoretical analysis focused on the shear capacity of the connections, and the main conclusions were drawn as follows:

(1) The failure mode of the shear connections was characterized by concrete failure near connectors and dowel bending, with no apparent bamboo failure. No significant difference in failure modes occurred between the CIP and assembled specimens.
(2) The load-slip curve of shear connections could be roughly divided into three stages. In the first stage, there was an approximately linear relationship between the load and the relative slip of the specimen. In this stage, the connectors effectively connected the bamboo and concrete, ensuring reliable shear transfer. In the second stage, the rate of load increase slowed down significantly, the slip rapidly increased, and the load-slip curve became nonlinear, mainly due to local cracking of the concrete leading to a reduction in shear stiffness. In the third stage, the curve started to decline, primarily due to local cracking and failure of the concrete block, yielding deformation of the connectors, and a decrease in the bonding strength between the connectors and concrete.
(3) The shear capacity of the assembled shear connections was similar to that of CIP shear connections, while the shear stiffness of the assembled shear connections was greater than that of the CIP shear connections. The dowel diameter of dowel shear connections was directly proportional to the shear capacity and inversely proportional to ductility.
(4) The slip distribution pattern of the shear connections was obtained based on the DIC-measured full-field displacement data. Before loading, the force transfer of the connectors was stable. During loading, the shear stiffness of the specimens continuously decreased. Prior to reaching the ultimate load, the slips on the left and right sides of the specimen were basically consistent, indicating no eccentric loading, and the connections on both sides were uniformly loaded. As the ultimate load approached, concrete failure at the dowel locations of the CIP dowel specimens resulted in relatively large local slips.
(5) Drawing from existing research on timber-concrete and steel-concrete shear connections, the shear mechanism of the connections was analyzed based on the characteristics of the specific connections under load to clarify the main factors affecting the shear capacity of the shear connections and the contributors to shear capacity. Based on Saulius’ theory, an accurate equation was proposed for calculating the shear capacity of dowel bamboo-concrete shear connections.

## Figures and Tables

**Table 1:** Parameters of the shear connections in the push-out tests.

Specimen Group	Specimen ID	Dowel Diameter (mm)	Fabrication Method	Number of Specimens
BDC16A	BDC16A-1/2/3	16	Assembly	3
BDC12A	BDC12A-1/2/3	12	Assembly	3
BDC16P	BDC16P-1/2/3	16	Cast-in-place	3

Note: B represents bamboo, D represents dowel, C represents concrete, 12/16 represents dowel diameter, A represents assembly, and P represents cast-in-place.

**Table 2:** Push-out test results of the BS-LC shear connections.

Specimen ID	Ultimate Load	Capacity of a Single Shear Connector	Maximum Slip	Stiffness
	*P*_max_ (kN)	1/4 *P*_max_ (kN)	Δ_max_ (mm)	*K* (kN/mm)
BDC16P	157.83	39.46	1.74	35.92
BDC16A	156.10	39.03	1.80	41.40
BDC12A	139.17	34.79	1.96	58.58

**Table 3:** Factors influencing the shear capacity of the representative connections with dowels.

No.	Reference	Calculation Equation	Parameters	Composite Member Type
1	Ceccotti [21]	$Q=0.23d^{2}\sqrt{f_{ck}E_{c}/\gamma_{v}}$	①⑥⑦	Timber-concrete
2	Saulius [22]	$\begin{array}{l} F_{u}=f_{ax}\cdot d\cdot t\cdot \left(\cot\alpha +\mu \right)+\\ f_{h}\cdot d\cdot t\cdot \left(1-\mu \cot\alpha \right)\left[\sqrt{2}\sqrt{\frac{2M_{y}}{f_{h}\cdot d\cdot t^{2}}\cdot \sin^{2}\alpha +1}-1\right] \end{array}$	①②③⑧	Timber-concrete
3	Lan [23]	$F_{u}=0.12\sqrt{f_{cw}\cdot f_{u}}\cdot d\cdot l_{E}$	①②③⑧	Timber-concrete
4	Cao [24]	$P_{u}=2.23\sqrt{\frac{f_{c}}{f_{u}}}\cdot f_{cw}\cdot d\cdot l_{E}$	①②③⑥⑧	Timber-concrete
5	Wang [25]	$F_{u}=0.14A_{s}{\left(\frac{f_{c}}{f_{u}}\right)}^{0.2}{\left(\frac{f_{cw}}{f_{u}}\right)}^{0.1}\frac{l_{E}}{d}$	①②③⑥⑧	Timber-concrete
6	Eurocode 4 [26]	$P_{Rd}=\frac{0.29\alpha d^{2}\sqrt{f_{ck}E_{cm}}}{\gamma_{v}}$	①②⑥⑦	Steel-concrete
7	GB 50017-2017 [27]	$N_{v}^{c}=0.43A_{s}\sqrt{f_{c}E_{c}}\leq 0.7A_{s}f_{u}$	①③⑥⑦	Steel-concrete
8	GB 50917-2013 [28]	$N_{v}^{c}=0.43\eta A_{std}\sqrt{f_{cd}E_{c}}$	①⑤⑥⑦	Steel-concrete
9	ANSI/AISC 360-16 [29]	$Q_{n}=0.5A_{sa}\sqrt{E_{c}{f_{c}}^{,}}\leq R_{g}R_{p}A_{sa}F_{u}$	①③⑥⑦	Steel-concrete
10	CSA S16-19 [30]	$q_{\mathrm{rs}}=0.50\phi_{sc}A_{sc}\sqrt{{f_{c}}^{’}E_{c}}\leq \phi_{sc}A_{sc}F_{u}$	①⑥⑦	Steel-concrete
11	Oehlers et al. [31]	$Q_{m}=5\cdot {\left(\frac{f_{cu}}{f_{u}}\right)}^{0.35}\cdot {\left(\frac{E_{cm}}{E_{s}}\right)}^{0.4}\cdot A_{sc}\cdot f_{u}$	①③④⑥⑦	Steel-concrete
12	Eurocode 5 [32]	$F(b)=f_{h,1}td[\sqrt{2+\frac{4M_{y}}{f_{h,1}dt^{2}}}-1]$	①②③⑥	Steel-Timber

Note: ① represents Dowel diameter, ② represents Dowel length, ③ represents Dowel tensile strength, ④ represents Dowel modulus of elasticity, ⑤ represents Longitudinal spacing of dowels, ⑥ represents Concrete strength, ⑦ represents Concrete modulus of elasticity, ⑧ represents Compressive strength of timber.

**Table 4:** Comparison of the shear capacity of bamboo-concrete connections with dowels (kN).

Specimen ID	Measured	Ceccotti [21]	Saulius [22]	Lan [23]	Cao [24]	Wang [25]	Eurocode 4 [26]	GB 50017-2017 [27]	GB50917-2013 [28]	AISC 360-16 [29]	CSA S16-19 [30]	Oehlers [31]	Eurocode 5 [32]
BDC16A	39.03	74.23	48.91	28.80	68.01	55.08	83.71	84.49	121.80	90.52	72.35	131.07	22.81
Measured/ Calculate	-	0.53	0.80	1.35	0.57	0.71	0.47	0.46	0.32	0.43	0.54	0.30	1.71
BDC12A	34.79	41.75	34.36	21.97	50.15	40.90	47.09	49.15	65.55	52.66	40.69	71.79	15.13
Measured/ Calculate	-	0.83	1.01	1.58	0.69	0.85	0.74	0.71	0.53	0.66	0.85	0.48	2.30
BDC16P	39.46	63.63	48.91	28.80	60.93	52.71	71.76	84.49	109.12	90.52	72.35	121.37	19.76
Measured/ calculated	-	0.62	0.81	1.37	0.65	0.75	0.55	0.47	0.36	0.44	0.55	0.33	2.00

**Table 5:** Comparison between the experimental and analytical shear capacities of connections with dowels.

Specimen ID	Measured (MPa)	Calculated (MPa)	Measured/Calculated
BDC16A-1	36.73	35.55	1.04
BDC16A-2	37.05	35.55	1.04
BDC16A-3	43.30	35.55	1.22
BDC12A-1	30.75	23.96	1.38
BDC12A-2	36.18	23.96	1.63
BDC12A-3	37.45	23.96	1.68
BDC16P-1	42.58	30.19	1.37
BDC16P-2	34.73	30.19	1.12
BDC16P-3	41.08	30.19	1.32
AVE	/	/	1.311
SD	/	/	0.235
COV	/	/	0.179

**Table 6:** Evaluation of the shear capacity equation for bamboo-concrete connections with dowels.

Reference	Specimen ID	Concrete Compressive Strength *f_h_* (MPa)	Dowel Diameter *d* (mm)	Actual Length of Dowel Embedded in Concrete *l_E_* (mm)	Ultimate Tensile Strength of Dowel *f_u_* (MPa)	Calculated Capacity of a Single Dowel *Q*_1_ (kN)	Experimentally Measured Capacity of a Single Dowel *Q*_2_ (kN)	Measured Capacity/Calculated Capacity
This work	BDC16A-1	55.96	16	40	600.59	35.48	36.73	1.04
	BDC16A-2	55.96	16	40	600.59	35.48	37.05	1.04
	BDC16A-3	55.96	16	40	600.59	35.48	43.30	1.22
	BDC12A-1	55.96	12	40	621.18	22.24	30.75	1.38
	BDC12A-2	55.96	12	40	621.18	22.24	36.18	1.63
	BDC12A-3	55.96	12	40	621.18	22.24	37.45	1.68
	BDC16P-1	44.92	16	40	600.59	31.08	42.58	1.37
	BDC16P-2	44.92	16	40	600.59	31.08	34.73	1.12
	BDC16P-3	44.92	16	40	600.59	31.08	41.08	1.32
Zhou [6]	D1	45	16	50	522.5	31.40	42.225	1.34
	D2	45	16	50	522.5	31.40	39.175	1.25
Wei [7]	D1	45	16	50	671.7	34.16	42.23	1.24
	D2	45	16	50	671.7	34.16	39.175	1.15
	D3	45	16	50	671.7	34.16	44.725	1.31
Hong [8]	BC1-1	35	8	60	400	10.63	8.95	0.84
	BC1-2	35	8	60	400	10.63	9.32	0.88
	BC1-3	35	8	60	400	10.63	9.09	0.86
	BC2-1	35	10	60	400	13.90	12.54	0.90
	BC2-2	35	10	60	400	13.90	10.99	0.79
	BC2-3	35	10	60	400	13.90	11.36	0.82
	BC3-1	35	12	60	400	17.57	21.41	1.22
	BC3-2	35	12	60	400	17.57	21.9	1.25
	BC3-3	35	12	60	400	17.57	21.61	1.23
	BC6-1	35	10	60	400	13.90	12.34	0.89
	BC6-2	35	10	60	400	13.90	11.25	0.81
	BC6-3	35	10	60	400	13.90	13.43	0.97
	BC7-1	35	10	60	400	13.90	12.06	0.87
	BC7-2	35	10	60	400	13.90	11.25	0.81
	BC7-3	35	10	60	400	13.90	11.55	0.83
	BC8-1	35	10	60	400	13.90	11.96	0.86
	BC8-3	35	10	60	400	13.90	10.69	0.77
Shan [9]	SC	57	18	100	600	68.53	64.5	0.94
Shan [10]	SC-1-16	38.6	16	80	428	34.27	44.6	1.30
	SC-1-18	38.6	18	80	431	40.24	47.9	1.19
	SC-1-20	38.6	20	80	445	47.05	49.7	1.06
	SC-1-22	38.6	22	80	469	54.97	51.2	0.93
	SC-2-18	38.6	18	80	838	47.33	56.2	1.19
Wang [11]	B3008	34.7	8	60	618.13	11.02	27.4275	2.49
	B3012	34.7	12	60	593.05	18.81	31.5075	1.68
	B3016	34.7	16	60	615.45	29.61	38.0825	1.29
	B5008	58	8	60	618.13	17.52	34.0725	1.94
	B5012	58	12	60	593.05	28.61	34.6	1.21
	B5016	58	16	60	615.45	42.87	43.815	1.02

## Data Availability

All the original data in this article can be obtained by contacting the corresponding author.
